# Sleep- and sleep deprivation-related changes of vertex auditory evoked potentials during the estrus cycle in female rats

**DOI:** 10.1038/s41598-024-56392-9

**Published:** 2024-03-09

**Authors:** Attila Tóth, Máté Traub, Norbert Bencsik, László Détári, Tünde Hajnik, Arpád Dobolyi

**Affiliations:** 1 In Vivo Electrophysiology Research Group, Department of Physiology and Neurobiology, Eötvös Loránd University, Pázmány Péter sétány 1/C, Budapest, 1117 Hungary; 2 Cellular Neurobiology Research Group, Department of Physiology and Neurobiology, Eötvös Loránd University, Budapest, Hungary; 3 Laboratory of Molecular and Systems Neurobiology, Department of Physiology and Neurobiology, Eötvös Loránd University, Budapest, Hungary

**Keywords:** Auditory evoked potentials, Estrus cycle, Female rats, In vivo electrophysiology, Sleep, Sleep deprivation, Vertex, Neuroscience, Physiology

## Abstract

The estrus cycle in female rodents has been shown to affect a variety of physiological functions. However, little is known about its presumably thorough effect on auditory processing during the sleep–wake cycle and sleep deprivation. Vertex auditory evoked potentials (vAEPs) were evoked by single click tone stimulation and recorded during different stages of the estrus cycle and sleep deprivation performed in metestrus and proestrus in female rats. vAEPs showed a strong sleep-dependency, with the largest amplitudes present during slow wave sleep while the smallest ones during wakefulness. Higher amplitudes and longer latencies were seen in the light phase during all vigilance stages. The largest amplitudes were found during proestrus (light phase) while the shortest latencies were seen during estrus (dark phase) compared to the 2nd day diestrus baseline. High-amplitude responses without latency changes were also seen during metestrus with increased homeostatic sleep drive. More intense and faster processing of auditory information during proestrus and estrus suggesting a more effective perception of relevant environmental cues presumably in preparation for sexual receptivity. A 4-h sleep deprivation resulted in more pronounced sleep recovery in metestrus compared to proestrus without difference in delta power replacement suggesting a better tolerance of sleep deprivation in proestrus. Sleep deprivation decreased neuronal excitability and responsiveness in a similar manner both during metestrus and proestrus, suggesting that the negative consequences of sleep deprivation on auditory processing may have a limited correlation with the estrus cycle stage.

## Introduction

Evoked potentials after sensory stimulation with various modalities are routinely measured from the vertex in humans^1–3^ as well as in animal models^4–7^. Middle-latency (10–100 ms) auditory evoked potentials (AEPs) can be recorded from midline vertex sites from both anesthetized^6^ and freely moving rats^4,8,9^ as vertex auditory evoked potentials (vAEPs). Components of vAEPs were relatively unaffected by bilateral lesions of the auditory cortex in rats^10^ implying that auditory cortex activity does not contribute significantly to the vertex maximal AEPs suggesting a largely independent generator of vertex AEPs from AEPs recorded from the auditory cortex in response to acoustic inputs transmitted by brainstem auditory centers^8^. vAEPs represent a relatively simple and efficient possibility to assess the changes related to sensory information processing of auditory stimuli due to the lateral location of the rat primary auditory cortex^11^ making the electrode implantation and recording difficult in freely moving rats^12,13^. Another aspect that supports the alternative investigation of vAEPs to assess auditory processing is the complicated definition of rat primary auditory cortex^14,15^.

Estrus (EST) cycle was found to affect a variety of physiological functions in female rats including social- and sexual behavior^16,17^, anxiety^18,19^ and sleep^20^. EST cycle is under strict and complex hormonal control with gonadal hormones playing a major role^21^.

Sleep was found to thoroughly affect different parameters of evoked potentials generated by various modalities of sensory stimulation reflecting the strong sleep–wake dependence of sensory functions as it was found in case of visual^22^, somatosensory^23^ and auditory stimulation^24,25^. Regarding AEPs, sleep-dependent patterns were described in male rats from both the primary auditory cortex^25,26^ and vertex^5^. However, the effect of reproductive cycle-related stages bearing a potentially strong influence on vAEPs during the sleep–wake cycle has not been reported yet. As the EST cycle was found to strongly affect the excitability of cortical neurons in female rats due to prominent hormonal effects^27^, it can be hypothesized that the EST cycle may strongly affect the sensory processing of auditory information in a sleep-dependent manner. Testing this possibility was the first aim of the present study.

Neuronal excitability can change not only as a function of the EST cycle, but also during sleep deprivation (SD)^9,28,29^. Reduced excitability can lead to reduced behavioral and cognitive performance characteristic to SD both in rat^30,31^ and human studies^32,33^. In a previous study, we reported decreased neuronal excitability and responsiveness measured by vAEPs during SD in male rats^9^. Therefore, the second aim of the present study was to extend these examinations to female rats. Different stages of the EST cycle were characterized by spontaneously changing homeostatic sleep drive^20^, cortical excitability and inhibition^27^ enabling the interpretation of vAEP changes during different conditions. We hypothesized that as a function of the different stages during the EST cycle, both restorative processes appearing during the recovery sleep after SD and SD-related excitability changes may be different in female rats further facilitating the understanding of the consequences of SD in females.

## Results

### Sleep–wake changes during the EST cycle

**Figure 1:**
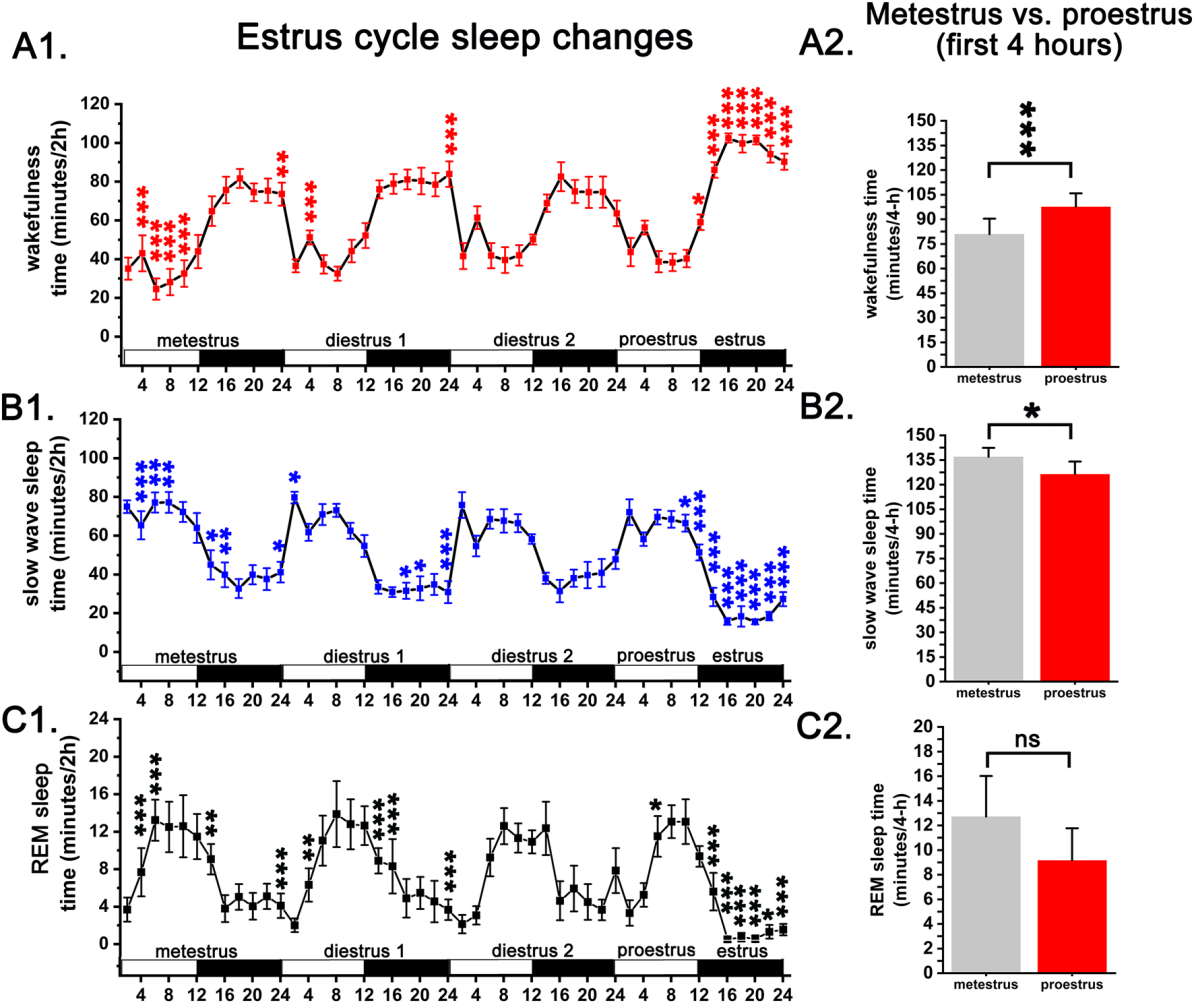
Sleep–wake changes during virgin EST cycle (n = 9). Panel A1, A2: W; panel B1, B2: SWS; panel C1, C2: REM sleep. Data were analyzed in 2-h long bins and expressed as minutes/2 h. MET duration was taken to be 24 h, DIE 48 h (DIE 1st day and DIE 2nd day) while PRO and EST 12–12 h, respectively. White and black bars at the X axis represent light- and dark phases, respectively. Asterisks  indicate significant deviation from the corresponding baseline (DIE2 day) value. Significance was tested with two-way ANOVA followed by Dunnett's multiple comparisons test. Significance levels: —*p* < 0.05; —*p* < 0.01; —*p* < 0.001. Data are expressed as mean ± S.E.M.

Sleep–wake changes were compared between the stages of virgin EST cycle using diestrus 2nd day (DIE2) as baseline. Metestrus (MET) was characterized by higher amount of slow wave sleep (SWS) and rapid eye movements (REM) sleep and lower wakefulness (W) in the light phase compared to the DIE2 baseline (Fig. 1A1, B1, C1). Increase in both sleep stages was also detected in the first third of the dark phase (Fig. 1B1, C1). MET vs. proestrus (PRO) sleep changes in the first 4-h period of the stages were also compared (Fig. 1A2, B2, C2). First 4-h of the PRO contained significantly more W and less SWS compared to MET (time X treatment, F (2, 48) = 20,7, *p* < 0,001 for W; *p* = 0,02 for SWS) (Fig. 1A2, B2) showing increased ongoing homeostatic sleep drive during MET compared to PRO. REM sleep amount did not show significant difference during the same 4-h period between the two EST cycle stages (Fig. 1C2).

DIE represented a stable stage regarding sleep with only few significant deviations in different time points during DIE1 compared to DIE2 baseline. These alterations were limited mainly to the initial and end period of the DIE1 period. At the beginning of the dark phase, lower level of W and higher level of both SWS and REM sleep could be seen (Fig. 1A1, B1, C1) while opposite changes were seen at the end of the dark phase compared to DIE2 baseline.

Strongest sleep changes occurred during the EST night. W strongly increased, SWS strongly decreased while REM sleep drastically decreased during this period resulting significant changes in all time points belonging to EST night in case of all three sleep–wake stages (Fig. 1A1, B1, C1).

### vAEP changes during the EST cycle

#### General characterization of vAEPs and general aspects of the vAEP analysis

**Figure 2:**
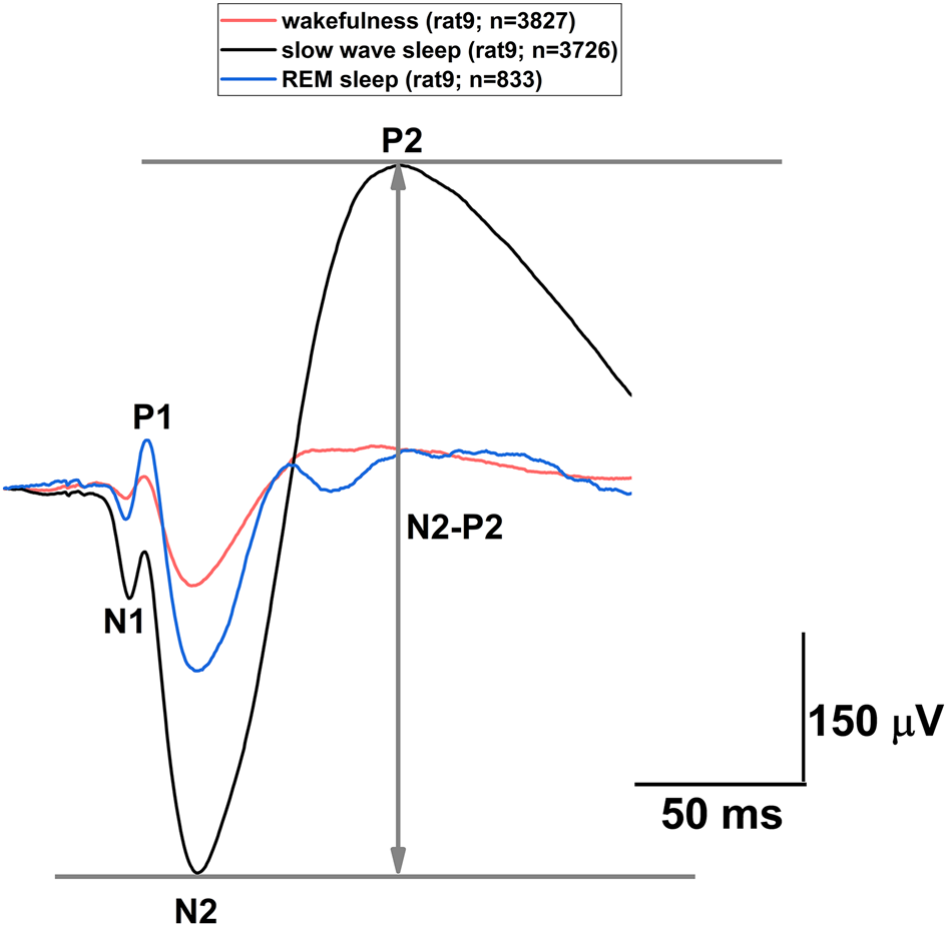
Traces of vAEPs and their analysed wave components averaged over 24 h of baseline DIE2 day during different sleep–wake states from a representative rat. Numbers in the upper legend indicate the number of averaged responses in the given sleep–wake stage. The fundamental difference in amplitudes is also clearly visible in the responses belonging to different vigilance levels.

In all cases, auditory stimulation with single clicks at a frequency of 0.1 Hz resulted in evoked potentials in the vertex region located in the occipital cortex in female rats. Although the evoked responses of each rat showed variability in the shape and amplitude of the individual components, the N1, P1, N2, P2 wave components could be observed and identified in all cases (Fig. 2). P1 wave was inconsistent in several cases and were therefore excluded from the analysis. As a consequence, N1–P1 component was also not analyzed.

**Figure 3:**
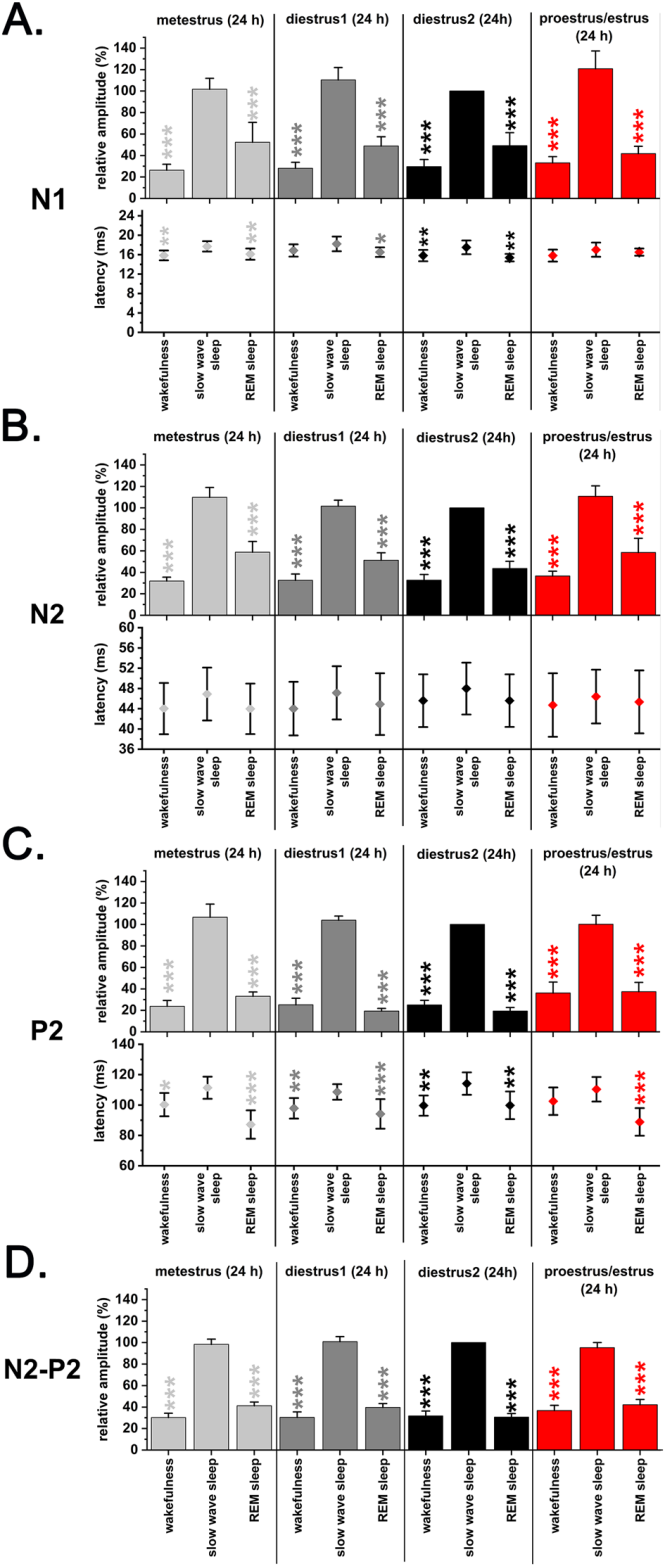
Sleep–wake-related changes in the amplitude and latency of the vAEP components during the EST cycle (MET, DIE1, DIE2, PRO/EST; n = 9). Panel A: N1; panel B: N2; panel C: P2; panel D: N2–P2. Relative amplitudes were calculated for W, SWS and REM sleep responses averaged in 24-h bins using DIE2 SWS average for 24-h as reference (100%). SWS was compared with both W and REM sleep during the same 24-h period. Significance was tested with two-way ANOVA followed by Dunnett's multiple comparisons test. Asterisks (*) indicate significant difference between W or REM sleep values compared to SWS values during the same day. Significance levels: —*p* < 0.05; —*p* < 0.01; —*p* < 0.001. Data are expressed as mean ± S.E.M.

**Figure 4:**
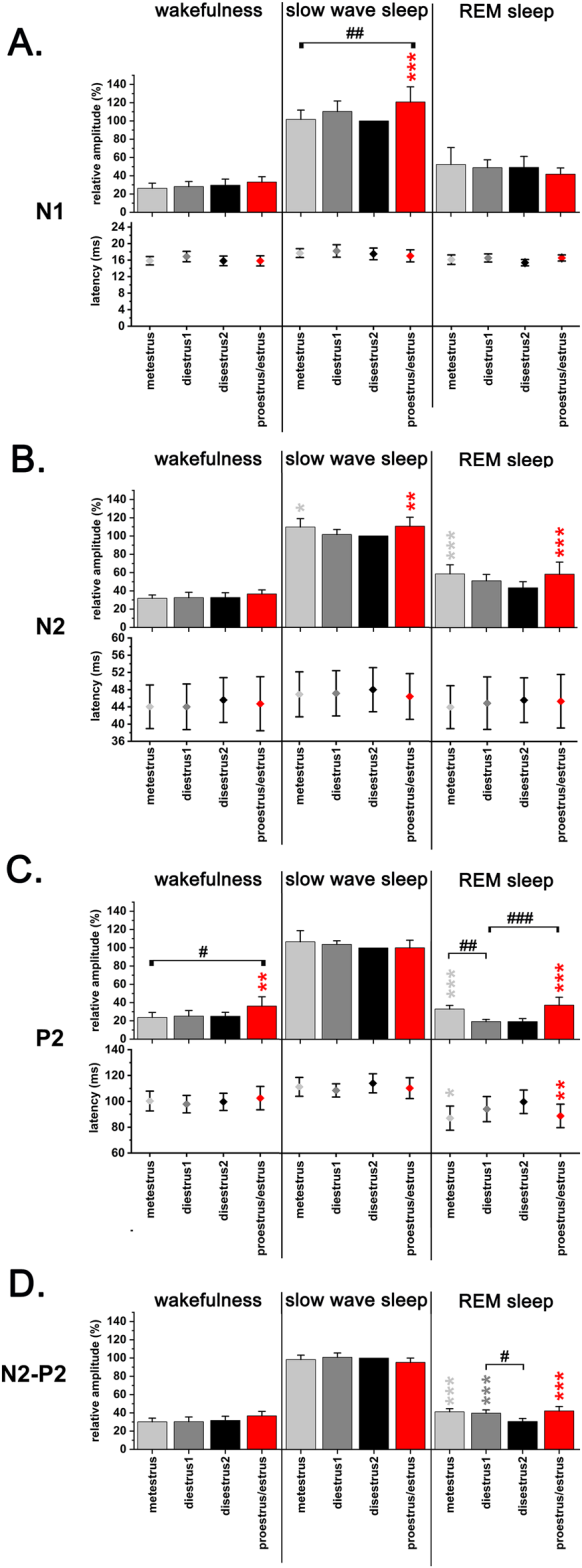
Changes of vAEP amplitudes and latencies during the EST cycle (MET, DIE1, DIE2, PRO/EST) by sleep–wake stages (n = 9). Panel A: N1; panel B: N2; panel C: P2; panel D: N2–P2. Relative amplitudes were calculated for W, SWS and REM sleep responses averaged in 24-h bins using DIE2 SWS average for 24-h as reference (100%). Significance was tested with two-way ANOVA followed by Dunnett's multiple comparisons test. Asterisks (*) indicate significant differences in the same sleep–wake stage compared to the DIE2 baseline. Hashtags (#) indicate significant difference between any of the values during the same sleep–wake stage in pairwise comparisons. Significance levels: —*p* < 0.05; —*p* < 0.01; —*p* < 0.001. Data are expressed as mean ± S.E.M.

**Figure 5:**
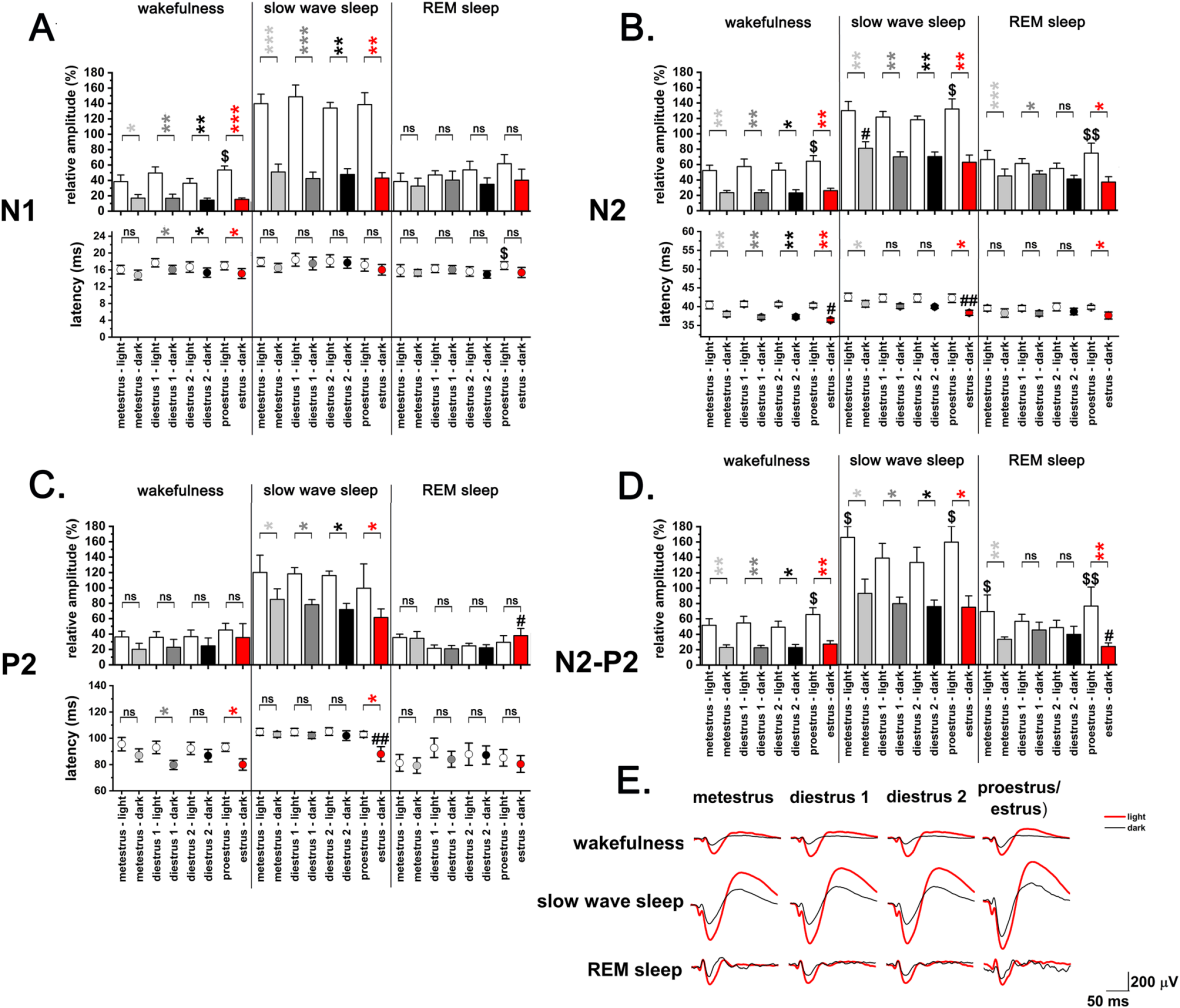
Changes of vAEP amplitudes and latencies during the EST cycle (MET, DIE1, DIE2, PRO/EST) by light–dark sleep–wake stages (n = 9). Panel A: N1; panel B: N2; panel C: P2; panel D: N2–P2. Panel E depicts light–dark averaged vAEP traces taken from a representative rat. Relative amplitudes were calculated for W, SWS and REM sleep responses averaged in 12–12-h light–dark phases using DIE2 SWS average for 24-h as reference (100%). Significance was tested with mixed-design ANOVA followed by Dunnett's multiple comparisons test. Asterisks (*) indicate significant light vs. dark differences in the same sleep–wake stage during the same day. Dollar-signs ($) indicates significant light vs. light differences in the same sleep–wake stage between different days compared to DIE2 reference. Hashtags (#) indicates significant dark vs. dark differences in the same sleep–wake stage between different days compared to DIE2 reference. Significance levels: —*p* < 0.05; —*p* < 0.01; —*p* < 0.001. Data are expressed as mean ± S.E.M.

VAEPs recorded during W, SWS and REM sleep were averaged over 24 h (Figs. 3, 4) and 12 h (light phase, dark phase; Fig. 5). No irregular EST cycles were observed in any of the rats in correlation with the long-lasting auditory stimulation. Thus, vAEP data were also analysed as a function of MET, DIE1, DIE2, PRO/EST stages of the EST cycle. DIE2 period served as baseline. Individual amplitude differences in different rats were normalized by setting DIE2 SWS as a reference (100%) for (i) responses evoked during other sleep–wake stages and (ii) other periods of the EST cycle. During the PRO/EST day, the light period was classified as PRO, while the dark period was classified as EST.

### Sleep–wake dependency of vAEP parameters during the EST cycle

#### Amplitude changes

For each day of the EST cycle, vAEP amplitudes for all analyzed response components (N1—Fig. 3A, N2—Fig. 3B, P2—Fig. 3C, N2–P2—Fig. 3D) differed significantly as a function of the sleep–wake stages analyzed inside the same day. Highest amplitude responses were seen in SWS while the lowest amplitudes in W while amplitudes during REM sleep were in-between.

#### Latency changes

Latencies of the different response components also followed a general trend in all 24-h periods analyzed. Longest latencies were seen during SWS while shorter latencies were seen during both W and REM sleep (Fig. 3A–C). Using these comparison (SWS vs. W; SWS vs. REM), utmost significant changes were seen in case of the P2 wave (Fig. 3C) and N1 wave (Fig. 3A).

### vAEP changes during the EST cycle by sleep–wake stages

#### Amplitude changes

24-h data were analyzed using an additional aspect. We also looked at how the response parameters varied in the same sleep–wake stage over the different days using the same data series as shown in Fig. 3.

Inside the EST cycle, PRO/EST day showed the most significant changes compared to DIE2 values as reference (Fig. 4). During W, PRO/EST P2 amplitude was significantly larger (time X treatment, F (6, 96) = 5.52; *p* = 0.002; Fig. 4C). During SWS, PRO/EST N1 (Fig. 4A) and N2 amplitudes (Fig. 4B) were significantly larger (time X treatment, F (6, 96) = 5,79; *p* < 0.001 for N1; time X treatment, F (6, 96) = 2.62; *p* = 0.007 for N2). During REM sleep, N2, P2 and N2-P2 amplitudes all showed significant elevation compared to the DIE2 reference (time X treatment, F (6, 96) = 2.62; *p* < 0.001 for N2; time X treatment, F (6, 96) = 5.52; *p* < 0.001 for P2; time X treatment, F (6, 96) = 7.45; *p* < 0.001 for N2-P2) (Fig. 4B,C,D).

MET contained the second most significant changes compared to DIE2 reference. N2 amplitude was significantly larger during SWS (time X treatment, F (6, 96) = 2.62; *p* = 0.02; Fig. 4B). During REM sleep, N2, P2, N2–P2 amplitudes were all significantly increased (time X treatment, F (6, 96) = 2.62; *p* < 0.001 for N2; time X treatment, F (6, 96) = 5.52; *p* < 0.001 for P2; time X treatment, F (6, 96) = 7.45; *p* < 0.001 for N2–P2) (Fig. 4B,C,D).

#### Latency changes

Latency changes were exclusively restricted to REM sleep and was seen only in case of the P2 component which showed significant decrease during both MET and PRO/EST compared to DIE2 reference (time X treatment, F (6, 96) = 1,86, *p* = 0,003 for MET; *p* = 0,01 for PRO/EST) (Fig. 4C).

#### vAEP changes during the EST cycle by light–dark sleep–wake stages

**Figure 6:**
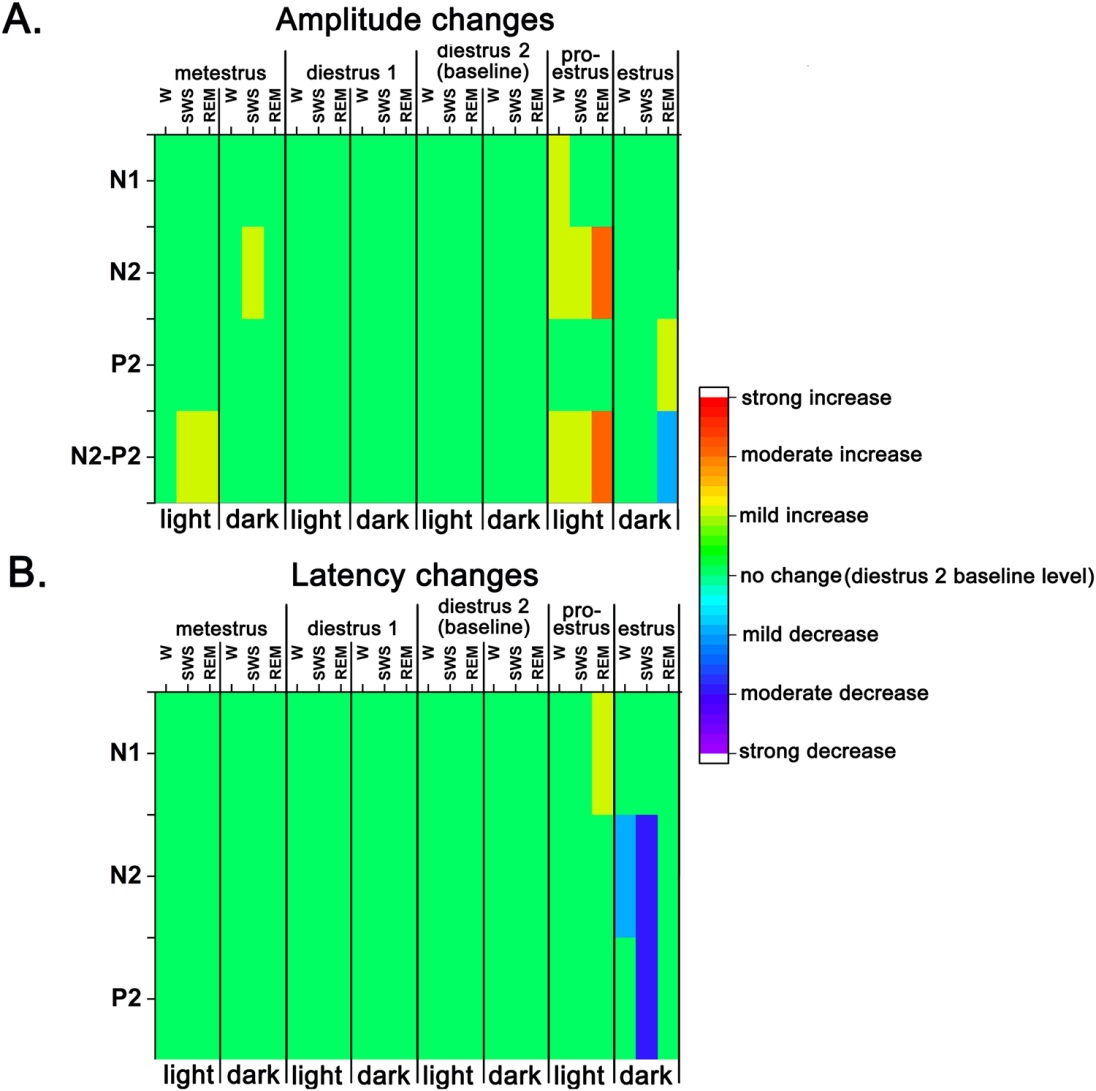
Summary of changes of vAEP amplitudes and latencies during the EST cycle (MET, DIE1, DIE2, PRO/EST) by light–dark sleep–wake stages. Panel A: amplitude changes, panel B: latency changes. Direction and magnitude of the changes was color-coded as mild- moderate- and strong changes are corresponding to significance levels provided by mixed-design ANOVA of *p* < 0.05, *p* < 0.01 and *p* < 0.001, respectively. Red: increase; blue: decrease.

VAEP changes in the same sleep–wake stage were also compared as a function of the 12–12 h light–dark cycle across the different stages of the EST cycle. Light–dark related vAEP amplitude- and latency changes between the different stages of the EST cycle compared to DIE2 baseline by sleep–wake stages are summarized and visualized as Fig. 6A and B, respectively.

#### Amplitude changes

In case of N1, N2 and N2-P2 components, most pairwise comparison (light vs. dark during the same sleep–wake stage, during the same day) showed significant difference in amplitudes favouring the light phase (Fig. 5A,B,D,E). In case of the P2 component, similar differences were seen only in SWS (Fig. 5C).

Light vs light and dark vs. dark values were also compared in the same sleep–wake stage, between the different days (MET, DIE1, PRO/EST vs. DIE2 as reference).

#### Amplitude changes in the light phase

In light vs. light comparisons, PRO/EST day showed the largest amplitudes in most cases during the light phase comparing PRO/EST data with data of other EST cycle stages (Fig. 5A,B,D).

In pairwise comparisons of PRO/EST N1, N2 and N2–P2 amplitudes vs. DIE2 corresponding references during the light phase, several comparisons resulted significant differences favouring the PRO/EST amplitude values. N1, N2 and N2-P2 values during W (mixed-design ANOVA: time, F (2,38, 19,0) = 41.1, *p* = 0.03 for N1; F (5, 40) = 41.1, *p* = 0.04 for N2; F (1,20, 9,64) = 14.1, *p* = 0.02 for N2-P2) were significantly higher during PRO/EST light phase compared to DIE2 reference ones. During REM sleep, N2 and N2–P2 components were elevated compared to DIE2 reference W (mixed-design ANOVA: time, F (5, 40) = 41.1, *p* = 0.001 for N2; F (1,20, 9,64) = 14.1, *p* = 0.004 for N2–P2).

MET also showed significant increases in light vs. light comparisons in some cases. N2–P2 amplitudes were enhanced during both SWS and REM sleep (mixed-design ANOVA: F (1,20, 9,64) = 14.1, *p* = 0.02 for SWS, *p* = 0.04 for REM sleep).

#### Amplitude changes in the dark phase

Compared to the amount of significant light vs. light amplitude differences, there were only a few significant changes in dark vs. dark comparisons. These changes were limited only to REM sleep in case of PRO/EST vs. DIE2 changes. P2 amplitude was significantly elevated while N2–P2 amplitude was significantly reduced during REM sleep (mixed-design ANOVA: F (5, 40) = 41.1, *p* = 0.03 for N2; F (1,20, 9,64) = 14.1, *p* = 0.04 for N2–P2) (Fig. 5C and D). MET N2 amplitude was significantly elevated during SWS (mixed-design ANOVA: F (5, 40) = 41.1, *p* = 0.04 for N2) (Fig. 5C).

#### Latency changes

In light vs dark comparisons, light phase latencies were longer compared to dark phase ones in case of all three response components with calculated latencies (Fig. 5A–C). In dark vs. dark comparisons, EST latencies were generally the shortest ones among the different phases of the EST cycle. Shortest EST latencies were seen during SWS in case of N2 and P2 components (mixed-design ANOVA: F (5, 40) = 6,53 *p* = 0,009 for N2, *p* = 0,007 for P2), during W in case of the N2 component (mixed-design ANOVA: F (5, 40) = 6,53 *p* = 0,04). Latency changes were summarized and visualized as Fig. 6B.

#### Total sleep deprivation during different phases of the EST cycle

**Figure 7:**
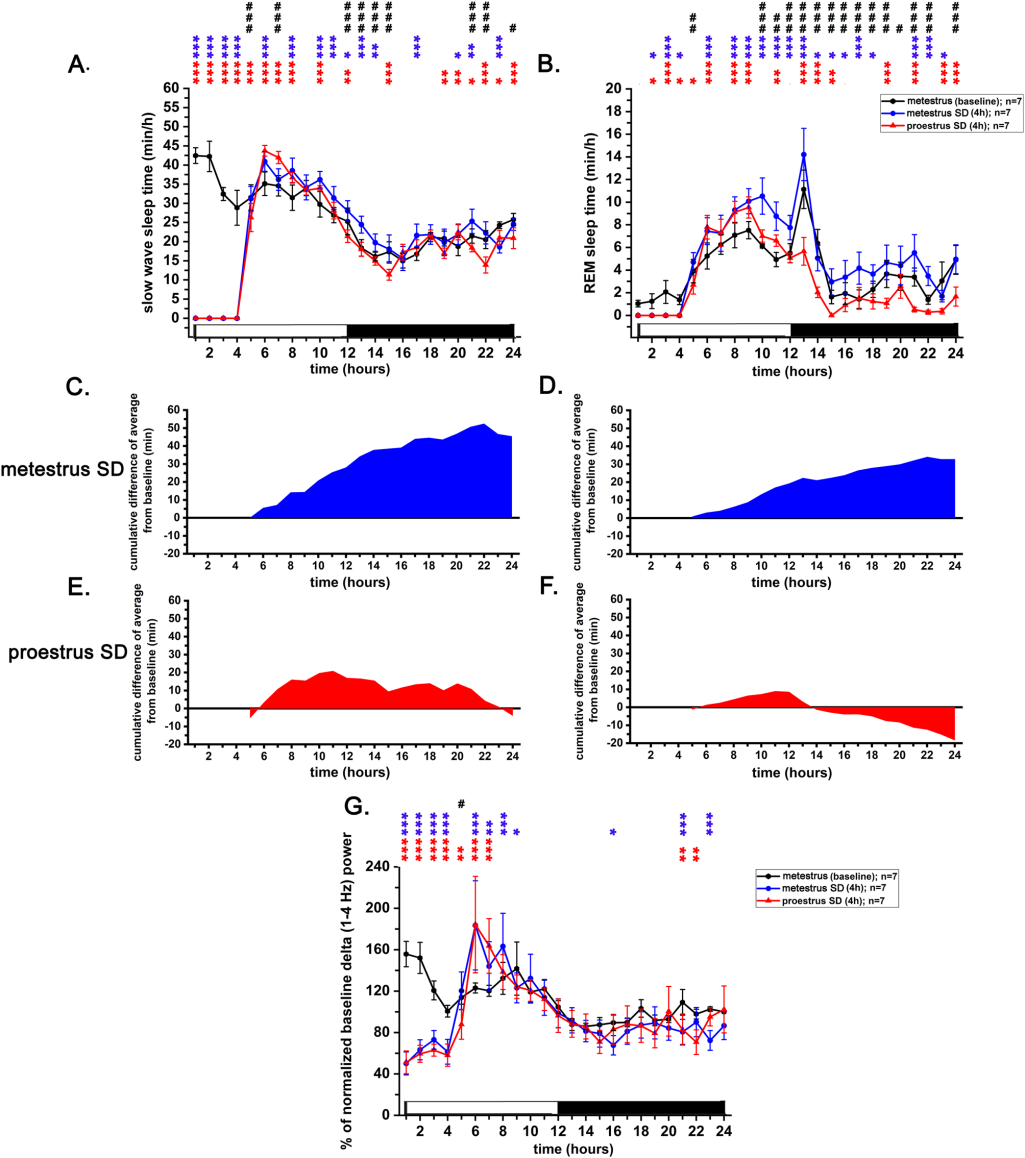
Effect of 4-h gentle handling total sleep deprivation on SWS, REM sleep and delta power performed during the MET- and PRO stages of the virgin EST cycle (n = 7). Panel A: SWS changes, panel B: REM sleep changes, panel C and D: cumulative difference of SWS and REM sleep from baseline, respectively, during MET SD; panel E and F: cumulative difference of SWS and REM sleep from baseline, respectively, during PRO SD, panel G: normalized delta (1–4 Hz) power changes during MET- and PRO SD sessions. Cumulative difference of the averages from the corresponding baseline average was depicted helping to assess the direction of the changes as well as the accumulating sleep deficit or excess regarding homeostatic sleep regulation. Data were analyzed in 2-h long bins and expressed as minutes/2-h for sleep data. White and black bars at the X axis represent light- and dark phases, respectively. Sleep and normalized delta power data of MET served as baseline. Red asterisks  indicate significant deviation from the corresponding baseline value in case of PRO SD. Blue asterisks  indicate significant deviation from the corresponding baseline value in case of MET SD. Hashtags (#) indicate significant difference between MET SD delta- vs. PRO SD delta power values in pairwise comparisons. Significance was tested with two-way ANOVA, followed by Dunnett's multiple comparisons test. Significance levels: —*p* < 0.05; —*p* < 0.01; —*p* < 0.001. Data are expressed as mean ± S.E.M.

Gentle handling 4-h SD sessions were applied at the beginning of the light phase during MET and PRO stages of the EST cycle (Fig. 7). During the SD sessions, total SD was achieved as no sleep occurred in any of the rats subjected to the SD procedure in any of the sessions. Undisturbed MET sleep data served as baseline for comparisons vs. MET SD and PRO SD. Effects of MET SD vs. PRO SD were also compared with each other.

#### Total sleep deprivation—effect on SWS

Both 4-h MET SD and 4-h PRO SD evoked strong homeostatic SWS response when the sleep was allowed again. Compared to MET baseline, strongest SWS response was seen between hours 6–9 in both cases (Fig. 7A). In this period, PRO SD sleep replacement was significantly higher compared to the MET SD one (Fig. 7A). However, in the remaining part of the light phase after hour 9 and in the dark phase, MET SD SWS replacement was significantly stronger in several time points compared to the PRO SD one. This resulted in a positive SWS balance at the end of the day for MET SD (~ 45 min compared to MET baseline; Fig. 7C). In opposition, a mild SWS debt was found in case of the PRO SD at the end of the day (~ 4 min; Fig. 7E).

Delta power (1–4 Hz) changes also reflected a less intense recovery after PRO SD compared to MET SD but the difference between MET SD vs. PRO SD delta power after the deprivation period showed significant difference only in hour 5 (Fig. 7G). A significant overshoot of delta power in the main period of recovery (hours 6–9) was seen in both cases. However, this delta rebound ceased earlier in hour 8 in case of PRO SD compared to MET SD (Fig. 7G).

#### Total sleep deprivation—effect on REM sleep

After total REM SD for 4-h, intense sleep replacement was seen between 6 and 12 h both after MET SD and PRO SD (Fig. 7B). Between hours 6–9, intensity of the recovery was not different during MET SD vs. PRO SD, but after this period, REM recovery became more intense after MET SD. This resulted in a complete recovery with positive REM sleep balance at the end of the day after MET SD (Fig. 7D), but not after PRO SD where a REM sleep debt existed at 24-h (Fig. 7F).

#### Effect of SD on VAEP parameters

**Figure 8:**
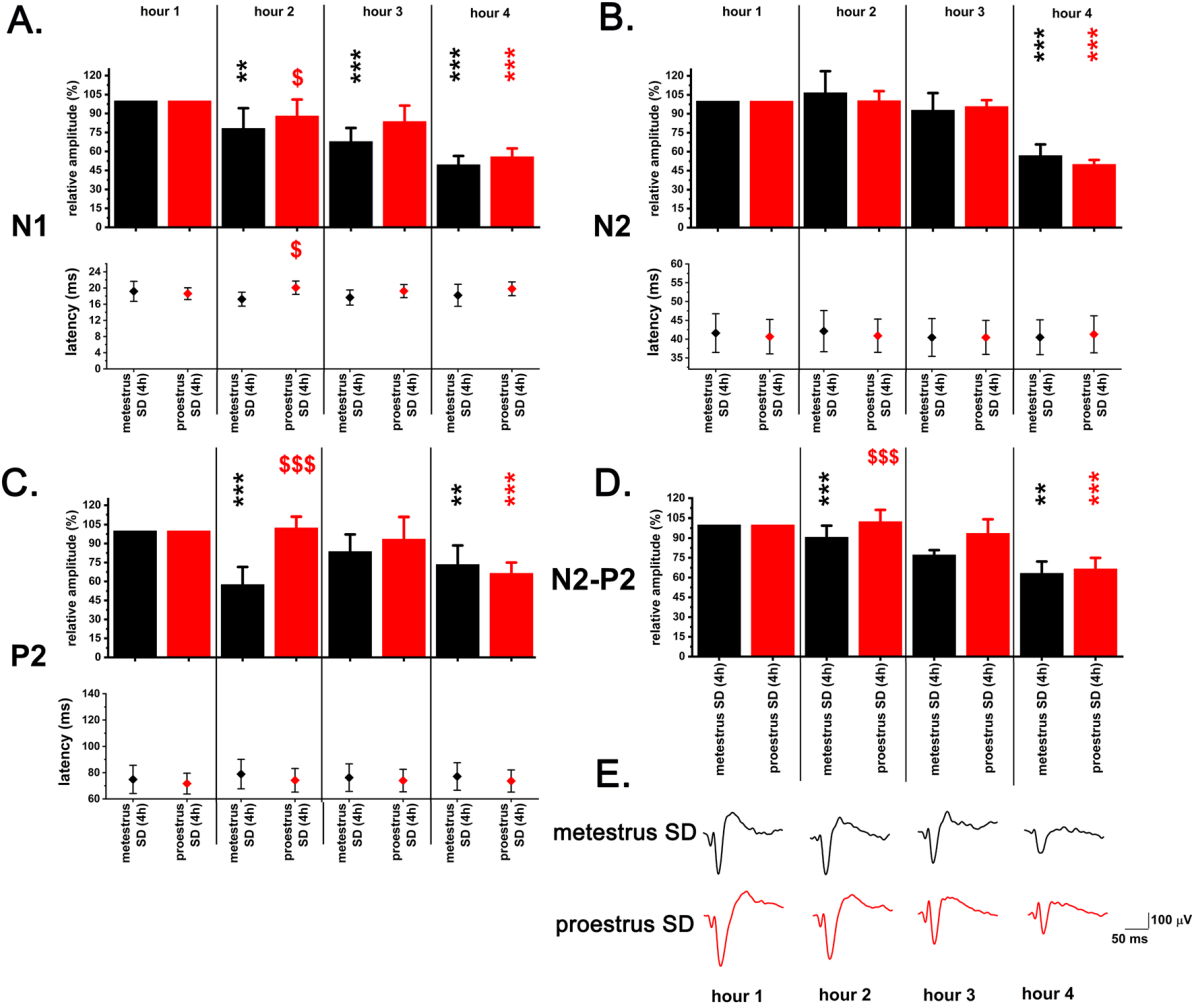
Amplitude- and latency changes of vAEPs during 4-h gentle handling total sleep deprivation performed in the MET- and PRO stages of the virgin EST cycle (n = 7). Panel A: N1; panel B: N2; panel C: P2; panel D: N2–P2. Panel E depicts 1-h averaged vAEP traces during MET- and PRO SD sessions taken from the same representative rat. Relative amplitudes were calculated using W vAEP data of the 1st SD hour as reference (100%) for data of later hours. Evoked potential data were averaged for 1-h bins and 1st hour of W data during SD served as baseline for statistical comparisons in case of both MET- and PRO SD sessions. Significance was tested with two-way ANOVA followed by Dunnett's multiple comparisons test. Black asterisks (*) indicate significant difference in SD hours 2,3 and 4 compared to 1st hour reference values in case of MET SD. Red asterisks  indicate significant difference in SD hours 2,3 and 4 compared to 1st hour reference values in case of PRO SD. Red dollar-signs  indicate significant difference in MET SD vs. PRO SD data in SD hours 2,3 and 4. Significance levels: —*p* < 0.05; —*p* < 0.01; —*p* < 0.001. Data are expressed as mean ± S.E.M.

SD caused a progressive decrease in the amplitudes of the vAEP components with strongest decrease seen in the 4th hour compared to 1st hour as baseline during both MET- and PRO SD (Fig. 8A–E). Negative polarity response components (N1 and N2) showed the strongest decrease (Fig. 8A, B). In hours 2–3, decrease of the amplitudes was smaller in case of PRO SD compared to MET SD (Fig. 8A,C,D), but the relative difference between PRO SD vs. MET SD disappeared in hour 4. In opposition of the amplitudes, response component latencies did not show significant decrease in any of the SD sessions performed during different stages of the EST cycle (Fig. 8A–D).

## Discussion

This is the first study, which comprehensively reports female rat vAEP changes (i) during the EST cycle as a function of the light–dark cycle and sleep–wake stages and (ii) during SD performed in different stages (MET and PRO) of the EST cycle. In order to separate the discussion to logical units and to compare the results with previous literature, we will first discuss sleep and vAEP changes during the EST cycle together with the possible physiological function of the vAEP changes found in these periods of the female reproductive cycle. Then, the discussion continues with vAEP findings during SD performed in different stages of the EST cycle.

### Sleep changes during EST cycle

Sleep–wake patterns seen during EST cycle in the present study were similar to previous experimental results from our laboratory^20^ and to literature data^34–36^. The most prominent sleep change in the EST cycle is the strong increase in W and decrease in SWS and REM sleep seen during the EST night^20,37,38^. During the light period of the subsequent MET day, loss of REM sleep and SWS are replaced in line with a homeostatic regulation^20,38^ appointing MET light phase as a period bearing the strongest spontaneous homeostatic sleep drive^20^.

Sleep changes during the EST cycle are related to changes in the underlying hormonal milieu of the cycle. In the case of estrogen (E2), the hormone has been found to significantly affect inhibitory processes in the cortex via its beta receptors by enhancing the excitability of parvalbumin-containing fast-firing interneurons, which led to an increase in inhibition both in vivo and in vitro^27^. In ovariectomized rats, supplementation with high doses of E2 led to shorter SWS episodes and more frequent awakenings during the dark period^39^.

### Sleep–wake stages strongly affected vAEPs during EST cycle

In the present study, we found strong sleep–wake variation in the amplitudes of all vAEP components analyzed. The highest amplitudes were seen during SWS, the lowest ones during W while REM sleep amplitudes were in-between.

Previous studies reported no consensus regarding this issue. In the study of Miyazato et al.^5^, P13 component of the vAEPs equivalent to N1 component in the present study was absent during SWS. P13 amplitude was similar during both W and REM sleep and amplitudes of higher-latency components showed no sleep–wake variation. In a study of Meeren et al.^25^, AEPs recorded from the auditory cortex changed in a similar fashion with the sleep–wake stages as in the present study. In a more recent rat study^40^ and also in the oldest^26^, auditory cortex AEP amplitudes were found to be the largest during SWS, but smallest during REM sleep while W in-between. In other studies, amplitudes during both W- and REM sleep were found to be similar^41,42^.

The abovementioned previous studies used male rats for vAEP or auditory cortex AEP recordings. Here we present the first time the sleep–wake dependence of vAEPs in female rats. Possible sex differences relating auditory processing, sleep–wake-related activity of the engaged networks and the significant variations in the technical implementation of the experiments (i.e. surface vs. deep layer cortical recordings, click vs. tone stimulation, different response component classifications and sleep scoring etc.) from study to study may help to explain the differences although in varying degrees. Albeit direct comparison between male vs. female vAEP data is not available to date, sex differences were found to bear an impact as seen in the latencies of brain-stem auditory evoked potentials in rats^43^.

In the present work, vAEP components reflecting both excitation (N1, N2)- and inhibition (P1, P2) on local field potential (LFP) level were always present independently from the sleep–wake stages. This is in opposition with a previous study reporting the absence of an excitatory component (P13) during SWS in male rats^5^. According to previous results, auditory stimuli can be decoded as summed postsynaptic potentials in the LFP^44^, our results show that this summation may occur in a stable way independently from the sleep–wake stage in female rats even if the exact network underlying the generation of vAEPs was not identified to date.

### vAEPs varied with light–dark cycle during EST cycle

In terms of the amplitude of the evoked responses, our results are in line with the classical knowledge in males that SWS has the highest degree of synchronization among the sleep–wake stages^45–47^ and the amplitude of the AEPs is proportional to the current neuronal network synchronisation. As a consequence, AEP amplitudes were remarkably higher during SWS compared to both W and REM phases^26,40,42^. In the present study, amplitudes of the vAEP components showed a pronounced light–dark differences, too. In case of all components, light phase responses had significantly higher amplitudes compared to dark phase ones. It was seen in case of all three sleep–wake stages with only a few exceptions appearing during W and REM sleep.

A light–dark dependent trend was also seen in latencies. Latencies were generally longer during the light phase, but shorter during the dark phase although it was less characteristic for the N1 component and for REM sleep overall. Latency changes may show correlation with amplitudes. In the light phase, evolution of the higher amplitudes required more time after stimulus onset resulting longer latencies. In the dark phase, smaller amplitudes could be evolved with shorter latencies.

Both the amplitude and latency changes can be seen as a function of the light–dark cycle as the level of SWS synchronization is higher during the light phase but lower during the dark phase according to previous data^46,48^.

For the lower latency N1 and N2 components, responses with higher amplitudes were seen in the light phase in case of all three sleep–wake phases. Also in this comparison, the highest amplitude responses were seen in the PRO/EST light phase, which was classified as PRO. The dependence of the responses on the current light–dark condition suggests that the synchronization during the light phase is higher under W than under dark.

Another factor which can determine vAEP amplitudes is excitability. At the level of individual neurons, excitability is higher during W than SWS^30,46^, but the low level of synchronization prevents the formation of high-amplitude responses at the LFP level. Thus, response amplitudes are regulated by the level of synchronization during SWS while amplitudes are also determined by the level of excitability during W.

To understand the effect of light–dark cycle on the vAEP, it is important to consider that vertex electrodes were positioned to the occipital cortex in this study and electrodes were located in the mediomedial secondary visual cortex^49^. Light–dark related firing of this area is not known to date, but baseline spiking during W was not altered in light vs. dark phases in the more lateral primary visual cortex^50^. However, spiking of cortical regular spiking neurons which are mainly pyramidal neurons representing the main cell type responsible for the current generation of cortical evoked potentials^44^ was found to be homeostatically regulated as a function of W length^46,50,51^. Longer W epochs characteristic to the dark phase induced higher frequency spiking compared to shorter W epochs seen during light phase^20^. Thus, although the firing may be more intense during dark W epochs, it may obstruct the summation of synaptic potentials appearing as decreased synchronization, which may explain smaller amplitudes and shorter latencies during dark phase W.

### vAEPs varied with the stages of the EST cycle—largest amplitudes and shortest latencies during PRO/EST

In the present study, across the stages of the EST cycle, largest amplitudes of vAEP components were seen during PRO (light phase) compared to DIE2 as baseline. PRO was the stage where largest amplitudes were present in most cases regarding different response components and sleep–wake stages. Amplitudes during MET were also larger in several cases compared to baseline, but remained below the PRO level in most comparisons. In turn, the most significant changes in latencies took place during EST night while latencies remained unchanged during MET.

Increased amplitudes observed both during MET and PRO can be explained differently. Gonadal hormones govern the EST cycle-related histological and physiological changes in female rats^21^. E2 level peaks during PRO, but during the EST night, level of E2 continues to decrease reaching a low level during MET^21,52^. E2 was found to be a potent modulator of neuronal excitability throughout the brain and can have both rapid and long-lasting effects on intrinsic and synaptic mechanisms of neuronal activity^53^ with significant region- and cell type-specific pattern^54,55^. E2 was found to strongly modulate inhibition in the cortex during the EST cycle by E2 β receptor-dependent mechanisms eliciting increased excitation of parvalbumin-containing fast-spiking interneurons in vitro and in vivo. Firing rate of fast-spiking interneurons was markedly lower during EST compared to MET and DIE while similar difference was not found in case of regular-spiking cells in vivo^27^*.* Interestingly, E2 increased the level of cortical inhibition without affecting excitation^27,56^. The contradictions regarding the presumed role of E2 can be resolved by the hypothesis that high E2 level may have a permissive role to enable ongoing neuronal activity suitable for the generation of high-amplitude evoked responses. This hypothesis may be applied even for PRO SWS with lower level of synchronization compared to MET due to the lower homeostatic sleep drive during PRO seen in the present study and also in our relevant previous study^20^. According to this hypothesis, enhanced vAEPs may be correlated with the increased homeostatic sleep drive and synchronization during MET but vAEP enhancement may be caused by E2 and/or other, yet unidentified neurochemical factors during PRO with reduced homeostatic sleep drive.

Among the vigilance stages, the highest amplitude excitatory vAEP responses (N1 and N2) were seen during PRO W. A recent study reported increased prefrontal-parietal LFP synchronization during PRO W compared to DIE W in the presence of a male^57^. However, the level of local synchronization in the vertex region can be more important shaping vAEP parameters. In a previous study of our laboratory, vertex gamma (30–48 Hz) LFP power was higher during PRO compared to DIE2 light phase^20^. LFP gamma activity reflects local neuronal synchrony in the high-frequency range and a hallmark of sensory processing in the cortex^58^, facilitating stimulus processing^59^. Based on these findings, increased synchrony reflected by enhanced LFP gamma power can be an explanation for increased vAEP responses during PRO.

### Functional implications of larger and faster-evolving vAEPs during PRO/EST

In rats, during EST and already during the onset of PRO, so-called EST behaviour can be observed, when the female becomes receptive to the sexual approach of the male and mating may occur^18^. This behavioral activation has been shown to be evoked by E2^60^ and governed by limbic structures^61^. PRO/EST was found to be associated with increased cognitive performance^62^ and reduced anxiety^18,63^. During PRO/EST, changes were found in sensory functions as well. This stage was characterized by increased visceral sensation including visceral pain sensitivity^64^ and elevated electrical shock threshold^65^.

Hearing range of rats is very wide extending from 500 Hz to 64 k Hz ultrasound at 60 dB sound pressure level^66^. Copulatory behavior during EST is associated with 22 kHz ultrasonic vocalizations^67^, used also for alarm^68^. Production of alarm ultrasonic vocalization was similar between female rats during both high- (PRO) and low (DIE1) ovarian hormone stages indicating no role of ovarian hormones in this type of acoustic communication^69^. Sonic vocalization plays a minor role in rat intraspecific communication. It can signal pain^70^ or can be a part of a defensive threat response to an oncoming predator^71^. In the present study, 4 kHz single clicks were applied as acoustic stimulation. This stimulation can represent external auditory information arriving form the environment and not related to any intraspecific social clue. Thus, more intense (increased vAEP amplitudes) and faster (reduced vAEP latencies) processing of sonic information during PRO/EST may function ensuring a more effective sensation of the environmental stimuli which may have a potential threatening context regarding the ecologically high-price reproduction in cooperatively-breeding rats^72^.

### SD during the different stages of the EST cycle resulted in stronger sleep recovery in MET

In our previous study, similar 4 h SD was performed during the EST cycle regardless of the actual EST cycle stage^20^. In the present study, we re-evaluated this topic by performing SD during different stages of the EST cycle. MET SD resulted in stronger SWS and REM sleep recovery compared to PRO SD in the present study. This was in line of the starting hypothesis that initial homeostatic sleep drive is higher during MET compared to PRO leading a stronger recovery in case of MET. SD was performed in the first third of the light period, but sleep recovery extended also for the dark period when spontaneous EST appeared in case of PRO SD. During EST, spontaneous sleep drive was reduced obstructing the replacement of sleep loss accumulated from the prior PRO. This mechanism was very characteristic in case of REM sleep resulted in REM sleep debt at the end of the day after PRO SD. These results suggest that SWS loss can be replaced during the EST cycle independently of the circadian time and EST cycle phase when the loss was accumulated. However, replacement of REM sleep can be EST cycle stage-dependent as REM sleep loss evolving during PRO is hampered to replace during the next EST. Thus, REM sleep loss can be tolerable on short run but long-term (96-h) total deprivation of REM sleep was found to disrupt the EST cycle causing constant DIE^73^. In case of 96-h REM SD, decreased release of the ovarian hormones, but increased release of corticosterone was found^73^ to cause permanent DIE. However, such strong hormonal effects may not evolve during 4-h SD.

### SD during the EST cycle decreased neuronal excitability measured by vAEPs

In our previous study, SD was found to decrease neuronal responsiveness and excitability in male rats^9^. In the present experiments, similar changes were observed in female rats. At the end of the SD period, vAEP amplitudes decreased in a similar manner both in case of MET SD and PRO SD yielding no significant difference between the effects of the different stages of the EST cycle. These stages are characterized by different homeostatic sleep drive, neurochemical milieu and possibly different initial excitability of the engaged networks at the beginning of the SD sessions. The fact that despite the initial stages were different but the results were similar suggests that negative consequences of the SD related to decreased neuronal excitability as seizure susceptibility^74–76^ and cognitive deficits^77–79^ may show limited dependency on the stages of the EST cycle in female rats.

## Conclusion

According to the present results, sleep–wake stages, light–dark phases, EST cycle and SD applied during different stages of the EST cycle all strongly affected vAEPs in cycling female rats. Largest amplitude vAEPs occurred during SWS while the smallest ones during W. Light phase vAEPs had higher amplitudes and longer latencies during all vigilance stages. During the EST cycle, largest amplitudes were found during PRO (light phase) while the shortest latencies were seen during EST (dark phase) compared to the DIE2 baseline. High-amplitude responses were also seen during MET with increased homeostatic sleep drive. More intense and faster processing of auditory information during PRO and EST suggesting a more effective sensation of relevant environmental cues probably to prepare for sexual receptiveness. A 4-h SD resulted in more pronounced sleep recovery in MET compared to PRO without difference in delta power replacement. It suggests a better tolerance of SD in PRO. As SD decreased neuronal excitability and responsiveness similarly during both MET and PRO, negative consequences of SD on auditory processing may show limited correlation with the EST cycle stage.

## Methods and materials

### Surgical procedure and housing

Experiments were performed on 9 female Wistar rats aged 10–11 weeks and weighing between 240 and 280 g at the time of the surgery. Electrode implantation was carried out under isoflurane anesthesia (Isoflutek 1000 mg/g, Alivira Animal Health Limited, Spain) applied using an RWD R540 small animal anesthesia machine (RWD Ltd, Shenzen, China). Isoflurane was mixed with air yielding a concentration of 1,4% and anesthesia was induced in a plexyglas chamber using 4 l/min flow. Then animals were supplied with anesthesia mask and placed into a stereotaxic frame (David-Kopf). The skin on the head was opened in the midline and muscles were retracted. During the surgery, isoflurane flow was set constantly to 1.5 l/min.

To record LFP activity, bipolar concentric electrodes (125 µm polyimide insulated stainless steel wire fixed in a 23G stainless steel tube) were implanted at the vertex (Bregma anteroposterior: − 4.5 mm, lateral: 2.0 mm) on both sides according to the rat stereotaxic atlas of Paxinos and Watson^49^. The used vertex coordinates located the electrodes in the mediomedial secondary visual cortex^49^. The tube touched the cortical surface, while the wire was inserted 1 mm below reaching layer 5. A stainless steel screw (diameter: 1.1 mm, Fine Science Tools) implanted into the bone over the cerebellum was used as reference and ground. To monitor electromyographic activity, a pair of Teflon-insulated stainless steel wire of 250 μm of diameter each was inserted into the neck musculature. All leads were soldered to a miniature connector prepared from a standard 50 × 2 connector strip (Precision) and the skull was covered with acrylic resin (Cranioplastic cement, Plastic One). Recording sessions started after 1–2 weeks of recovery. The surgery and electrode implantation was similar to that published earlier by our laboratory^20,80,81^. Following surgery, the animals were kept warm, and painkiller (50 mg/kg metamizole, i.p.) was administered for 3 days.

Rats were kept in a LD12:12 cycle (lights on at 10:00) and were housed in individual cages located in a sound-attenuated room throughout the whole experiment. The cages were prepared from clear Plexiglas cylinders (height: 330 mm, diameter: 300 mm). Water and standard laboratory chow were available ad libitum.

Rats were connected to the recording apparatus three days before the first recording session to allow habituation to the recording situation. Flexible flat cables connected the rats to swivels fixed above the large Plexiglas cylinders during the recordings. Cables were folded to a zigzag shape with a rubber string running in the middle to provide free movements of the rat. The experimental setup was similar to that published earlier by our laboratory^20,80–83^ and others^84^.

Experiments were carried out in accordance with the Hungarian Act of Animal Care and Experimentation (1998, XXVIII) and with the directive 2010/63/EU of the European Parliament and of the Council of 22 September 2010 on the protection of animals used for scientific purposes. Experimental protocols were approved by the Ethical Board of Eötvös Loránd University. All the performed procedures and all the reported data were in accordance with the ARRIVE guidelines.

### Experimental timeline

Virgin EST cycle was characterized by 120-h long recordings running without interruption covering a whole EST cycle. During the recordings, the animals were not exposed to any external stimuli or interventions.

Recording sessions during SD applied in different stages of the EST cycle lasted for 24 h. Before the recording sessions started, rats were habituated for the chronic stimulation conditions applying 72-h continuous auditory stimulation without recordings. An idealized experimental timeline is presented as Supplementary Fig. S1.

### Vaginal smear cytology

Vaginal smear sampling was performed at the light onset daily both in case of the 120-h recordings and 24-h SD sessions. Before the recordings had started, rats were habituated for the sampling procedure to avoid non-specific behavioral activation which may alter homeostatic sleep regulation. Vaginal smear samples were dried to glass slides and stained by 1% methylene blue solution. Stages of the EST cycle were identified according to standard histological criteria^85^. MET was characterized by cornified epithelial cells and leukocytes. DIE smear contained nucleated epithelial cells, leukocytes, mucus and cellular debris. PRO smear was consisted of mainly nucleated epithelial cells while EST smear was dominated by anucleated cornified epithelial cells.

### EST cycle staging

Length of the EST cycle was found to be 96 h long. MET duration was taken to be 24 h, DIE 48 h (1st day—DIE1; 2nd day—DIE2) while PRO and EST 12–12 h, respectively, according to the conventional literature values^86^ and our previous study examining cycling female rats^20^. According to the hormone release profiles, PRO occurs during the light phase while EST mainly involves a single dark phase in case of the generally used LD12:12 lighting conditions^21,87^.

EST cycle staging was performed using different ways of information as described previously^20^. First, vaginal smear cytology, second, the very characteristic REM sleep reduction seen during one dark phase, or rarely, on two dark phases in the 120 h recordings involving five dark phases. EST night REM sleep reduction was seen for each rat during the control EST cycle recordings.

### Total sleep deprivation

All total SD session started at lights on, lasted for 4-h and were performed by the gentle handling method. Whenever the animals appeared drowsy or slow waves emerged in the LFP, the animals were mildly disturbed by moderate noise, fragrant soap, cardboard pieces, bedding materials from other animal or by introducing fresh water or fresh food. During the gentle handling method, the animals were never touched^88^. After the deprivation period, recordings were continued for further 20 h without interruption.

EST cycle phase was determined by vaginal smear cytology at the morning of the SD session. One SD session was performed during MET and another during PRO using the same pool of rats (n = 7). At least 3 days elapsed between two consecutive SD sessions in case of each involved rat. MET was taken as baseline for comparison.

### Electrophysiological recording and acoustic stimulation

Auditory click stimuli with 500 µs duration and 4 kHz frequency were delivered every 10 s by a piezoelectric speaker attached to the top of the cage, approximately 30 cm high above the animal. Stimulus intensity was set empirically, to ensure that no startle response or any twitch of the auricles was induced by the click. The intensity was assessed to be about 60 dB also depending on the position of the animal.

Signals were amplified (2000x) and filtered (0,3 Hz–2 kHz) (Elsoft BT), then digitalized at a 16-bit resolution by an analogue-to-digital (A/D) converter card (Labview, National Instruments, Austin, TX, USA). The A/D sampling frequency was set to 2048 Hz (a power of 2) to facilitate Fast Fourier analysis.

### Data analysis

#### Sleep scoring

Sleep stages were scored using custom-made semi-automatic software. The program enabled visual inspection of the recorded signals, digital filtering, and spectral analysis of the LFP curves. Power spectra were calculated using the Fast Fourier Transformation algorithm for all consecutive 4-s periods from all recordings. Power was integrated in the delta (0.5–4 Hz), theta (4–10 Hz), alpha/sigma (10–16 Hz), beta (14–30 Hz) and gamma (30–48 Hz) frequency ranges and the ratio of the theta/delta power was determined. Electromyography data was also processed using the Fast Fourier Transformation method and the total power (variance) was calculated in the 5–48 Hz range.

Epochs containing movement artefacts (high delta power and high electromyographic activity variance; less than 1% of the recordings) and REM sleep epochs (low delta power, high theta/delta ratio, and low muscle tone) were manually selected in all recordings by visual inspection of the LFP and electromyography signals^9,20,81,89,90^. Epochs containing artefacts were excluded from further analysis (less than 1% of the baseline recording and recovery and around 5% during SD).

In the present experiments, delta power and electromyographic activity thresholds were set individually for each rat by visual inspection of the raw LFP and electromyography data from baseline recordings (DIE2 day for EST cycle vAEP analysis, MET for SD vAEP analysis). These objective thresholds were then used to score recordings obtained during the non-baseline stages of the EST cycle (MET, DIE1, PRO/EST) and for SD sessions performed during MET and PRO. Epochs in which delta power was above and electromyographic activity value below these thresholds, respectively, were marked as SWS, while epochs with lower delta power or higher electromyographic activity as W. Scoring was performed by a semi-automated method that had been published earlier^89^ using the slow wave content of the LFP as a main parameter for sleep scoring as delta power (˂ 4 Hz) is closely and inversely related to the level of cortical arousal^91^. The software and delta power based scoring method was used in several studies published by our laboratory^20,80–82,90,92,93^. Raw hypnograms were smoothed, i.e. every 32-s period was assigned to the dominant sleep–wake stage^94^.

#### vAEP analysis

vAEP data were recorded from layer 5 using the bipolar concentric electrodes. To obtain vAEP data, 200-ms LFP epochs after the stimulus onset were cut from the raw LFP curves. VAEPs were found to be strongly influenced by the vigilance level, especially in the domain of longer latency components^5,95^. VAEPs were averaged as a function of the sleep–wake stages using the hypnogram generated by sleep scoring. Each bin containing a vAEP response was assigned to the concurrent vigilance stage. Then vAEPs were averaged in 12- and 24 h long blocks separately by sleep–wake stages. In case of SD sessions, vAEP data were averaged in consecutive 1-h blocks. Because during the SD procedure the rats were mostly awake, the majority of the vAEPs was selected for averaging. Ratio of the excluded vAEPs never exceeded 15% even in hour 4 of SD when sleep pressure was high.

Short- and mid-latency components (10–100 ms) of the vAEPs were analyzed. Latencies of the first two negative and positive peaks (N1, P1, N2 and P2) and their peak-to-peak amplitude (N1–P1 and N2–P2) were averaged in 1-h blocks (SD) and/or 12- or 24-h blocks (EST cycle). P1 wave was inconsistent in several cases and were therefore excluded from the analysis. As a consequence, the N1-P1 values were also not analyzed.

For the amplitude analysis of the different response components (N1, N2, P2, N2-P2), normalization was applied. 24-h average of amplitudes seen during SWS of the baseline DIE2 day was set as reference (100%). Similar normalization was applied for each response component and in each rat separately. Relative amplitudes were expressed as percentage of the reference and were calculated for vAEPs belonging to other sleep–wake stages (i.e. W and REM sleep) and/or other time windows (1-h or 12-h) as well.

#### Delta power analysis

LFP power values in the delta (1–4 Hz) range were averaged for 60-min long periods and normalized using the data of the defined baseline day for SD sessions (MET day of the EST cycle). Grand average of the power values of all hours and frequency bands (delta, theta, alpha/sigma, beta, gamma) of the baseline day was calculated as ’normalization factor’. Then each of the power values belonging to any time point were divided by the same ’normalization factor’ in case of both the baseline and the SD recordings. Normalized values then summarized for 60-min long periods. After that, values of the 1-h periods of the baseline day were averaged separately in the different bands. Actual values of the 1-h periods were divided by this daily average and expressed as average percentage by frequency bands. Normalized values belonging to the treatment days were also divided by the baseline daily average separately for each frequency bands and expressed as baseline percentage. Normalized LFP power data were calculated for each frequency band but only the data of the delta (1–4 Hz) band is shown in the present study as the most relevant LFP activity indicating the changes of homeostatic sleep pressure^96^.

### Statistical analysis

Data were analyzed by two-way analysis of variance (ANOVA) followed by Dunnett's post-hoc test in 1-h bins (SD sleep and vAEP data), 2-h bins (EST cycle sleep data), 4-h bins (MET and PRO sleep data for the first 4-h of the stages) or 24-h bins (vAEP data). DIE2 values served as baseline/reference for both sleep- and vAEP data. During the EST cycle, low and steady levels of both ovarian and pituitary hormones during DIE2^97^ made this stage ideal to be taken account as baseline similarly to our previos study using the same female rat model^20^.

Mixed-model (split-plot) ANOVA has higher statistical power compared to ordinary two-way ANOVA when analysed datasets contain missing values in a randomized fashion^98^. In the present study, it was the case for one rat which had no REM sleep during the EST night. According to this, mixed-design ANOVA with Greenhouse–Geisser correction was used for 12-h bin light–dark vAEP data.

For the 12-h data, light vs. dark differences but also light vs. light and dark vs. dark differences were also checked. Statistical tests were applied separately for each different response component amplitudes (N1, N2, P2, N2–P2) and latencies (N1, N2, P2), respectively. Statistical tests were performed by parameters using whole datasets containing all relevant W, SWS and REM sleep data. In most cases, values of a given parameter were compared by vigilance stages across different days/EST cycle stages. All tests were two-tailed and *p* < 0.05 was accepted as the lowest limit of significant difference.

The term „time X treatment” reflects the interaction between the factors indicated by the two-way ANOVA. The term “treatment” denotes different stages of the EST cycle (MET, DIE1, DIE2, PRO/EST). 

Significance levels in the figures are indicated: *,$,#—*p* < 0.05; **,$$,##—*p* < 0.01; ***,$$$,###—* p* < 0.001. Data are shown as mean ± S.E.M. (standard error of the mean) on all figures. Statistical analysis was performed using Prism 8.0 (GraphPad Software, San Diego, USA). Data were plotted in Microcal Origin 2018 (OriginLab Corporation, Northampton, USA). Final editing was performed using Adobe Photoshop CC.

### Supplementary Information

Supplementary Information 1.

Supplementary Figure S1.

## Data Availability

All data analyzed and presented in this work are available from the corresponding author upon reasonable request.

## Abbreviations

AEP: Auditory evoked potential
DIE: Diestrus
E2: Estrogen
EST: Estrus
LFP: Local field potential
MET: Metestrus
PRO: Proestrus
REM sleep: Rapid eye movements sleep
SD: Sleep deprivation
SWS: Slow wave sleep
vAEP: Vertex auditory evoked potential
W: Wakefulness
