# Supplementary material for: Sleep- and sleep deprivation-related changes of vertex auditory evoked potentials during the estrus cycle in female rats
Source: Sci Rep. 2024 Mar 9;14:5784. doi: 10.1038/s41598-024-56392-9 (PMC10924932; doi:10.1038/s41598-024-56392-9)
Supplement: Supplementary file 1 — Supplementary Information 1. [file 41598_2024_56392_MOESM1_ESM.docx]

Supplementary Figure S1. Timeline of the experiments. Note that the scheme shows an ideal case, some experiment lasted longer because EST cycles were only partially synchronized between the rats. Due to this, SD sessions during MET and PRO covered not one, but two consecutive EST cycles. 4-h SD sessions started at the onset of the light phase both during MET and PRO.

Horizontal white and black bars represent 12-12 hours consecutive light- and dark phases, respectively. During the habituation period, rats were habituated to both the continuous auditory stimulation and daily vaginal smear sampling performed at the onset of the light period.
