# Supplementary figures and images for: Sleep- and sleep deprivation-related changes of vertex auditory evoked potentials during the estrus cycle in female rats
Source: Sci Rep. 2024 Mar 9;14:5784. doi: 10.1038/s41598-024-56392-9 (PMC10924932; doi:10.1038/s41598-024-56392-9)

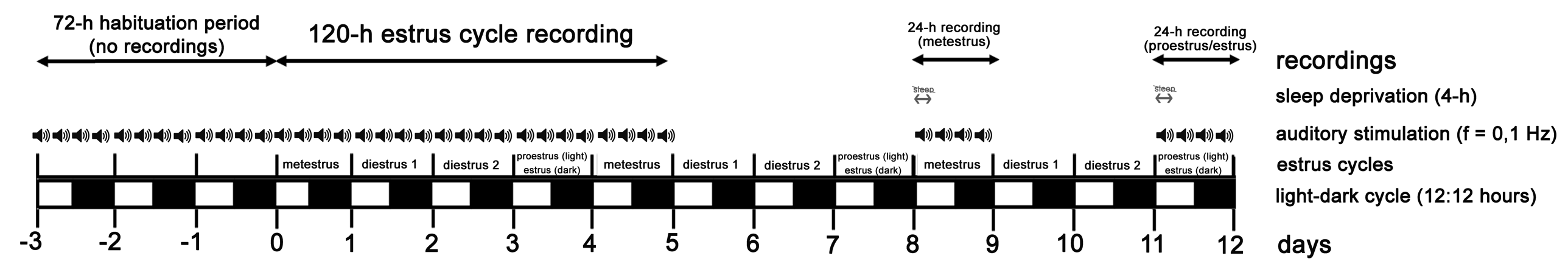

Supplement: Supplementary file 2 — Supplementary Figure S1. [file 41598_2024_56392_MOESM2_ESM.tif]
